# Supplementary material for: Soccer clubs as avenues for gender transformative socialization of adolescent boys in Cape Town and Mthatha, South Africa: A qualitative study
Source: PLoS One. 2023 Feb 2;18(2):e0280932. doi: 10.1371/journal.pone.0280932 (PMC9894412; doi:10.1371/journal.pone.0280932)
Supplement: S1 File — (DOCX) [file pone.0280932.s001.docx]

# **Soccer clubs as social avenues for engaging adolescent boys in gender-transformative and HIV prevention programming.**

# **IDI guide with coaches**

**Background information**

Please may you share with me your age?

Please tell me about your coaching background and training, and how long you have been coaching.

Please tell me about what the job of coaching adolescent boys entails.

Probe: what are the ages of the adolescent boys you train in your club?

Probe: How many adolescent boys do you currently train in your club?

Probe: Where do you train them and how often do you train them in a week?

Probe: who are the rest of the club management and support team?

**Background information on the players**

Please may you tell me about the background of players in your club

Probe: where are they from?

Probe: what are their ages?

Probe: how do they get to the training venue and to the games?

Probe: How would you describe their socio-economic backgrounds?

Probe: what financial support does your club have?

What has been your experience of working with adolescent boys as soccer players in your club?

*Probe:* As a coach, what roles do you find yourself playing in the lives of the adolescent boys that you train in your club?

What do you understand to be the role of soccer coaches working with adolescent boys in their club?

Please share your thoughts and experiences about the social challenges that adolescent boys in your club face.

*Probe:* Please share stories of how social challenges have affected the lives of adolescent boys in your club.

*Probe:* How have you or other coaches in your club responded to the social challenges of adolescent boys in your team face?

*Probe:* What kind of support has your team offered adolescent boys who were facing social challenges?

*Probe:* What kind of support does your team needs to be able to adequately support adolescent boys to address their social challenges?

As a coach, could you share with me instances where you have talked with adolescent boys in your club about sexual reproductive health and rights issues?

Please tell me your experiences where you got to know or suspected that some adolescent boy(s) where experiencing sexual health problems.

*Probe:* In those instances, what help did the boy(s) access to address their problem?

Probe: Share with me stories of how sexual health problems have affected the lives of adolescent boys in your club.

*Probe:* How have you or other coaches in your club responded when adolescent boys in your club faced sexual health problems?

*Probe:* What kind of support has your team been able to offer adolescent boys going through social and health challenges?

*Probe:* What kind of support does your team need to be able to adequately support adolescent boys to address their sexual health problems?

In your view, what kind of support do adolescent boys in your team need to enable them to better navigate the adolescent stage, protect themselves from STIs and HIV and adopt gender-equitable masculinities?

In your experience of coaching adolescent boys in this community, what are their hopes and aspirations regarding the kind of men (masculinities) they want to be?

In this community, what do adolescent boys hope and aspire to achieve as boys/men?

Could you share with me instances where you or other coaches in your club engaged soccer players (adolescent boys) in issues related to gender equality?

*Probe:* How was that experience for you as a coach?

*Probe*: How confident are you to talk about gender equality issues with adolescent boys in your club?

*Probe*: In terms of knowledge and communication skills, how equipped do you feel you and other coaches are to talk about gender equality issues with adolescent boys in your club?

*Probe*: What challenges do you envisage for you and other coaches in your club in engaging adolescent boys in gender equality issues?

Please tell me about the times where you or other coaches in your club engaged soccer players (adolescent boys) about issues related to dating, sexual relationships, STIs and HIV?

*Probe:* How was that experience for you as a coach?

*Probe*: How confident are you to talk about dating, sexual relationships, STIs, and HIV with adolescent boys in your club?

*Probe* In terms of knowledge and communication skills, how equipped do you feel you and other coaches are to talk about dating, sexual relationships, STIs, and HIV with adolescent boys in your club?

*Probe*: What challenges do you foresee for you and other soccer coaches in your club in talking to adolescent boys about dating, sexual relationships, STIs and HIV?

*Probe*: What else do you think should be done to support adolescent boys to make healthier choices with regard to dating, sexual relationships, STIs, and HIV?

How do you balance the teaching of lessons on the field that can apply to life off the field?

Please may you share with me which strategies of engaging adolescent boys (in the topic above) have worked well and why?

Please may you share with me which strategies of engaging adolescent boys (in the topics above) have not worked so well and why?

What does the future look like for coaching adolescents in this environment and why?

Do you have any questions for me?

**End of the interview**
