# Supplementary material for: Soccer clubs as avenues for gender transformative socialization of adolescent boys in Cape Town and Mthatha, South Africa: A qualitative study
Source: PLoS One. 2023 Feb 2;18(2):e0280932. doi: 10.1371/journal.pone.0280932 (PMC9894412; doi:10.1371/journal.pone.0280932)
Supplement: Attachment — Submitted filename: Response to Reviewers.docx [file pone.0280932.s002.docx]

Dear Prof Webster Mavhu,

A sincere thank you to you for giving us the opportunity to have our paper entitled “The potential of soccer clubs as avenues for gender transformative socialization of adolescent boys in Cape Town and Mthatha, South Africa: findings from a qualitative study’ revised and resubmitted to PloS One.

We considered the reviewers’ comments and feedback and yours to be very valuable and have summarised the amendments in the table below.

All the comments are listed and responses from the authors are given accordingly.

| **Editor** | **Response from authors** |
| --- | --- |
|  |  |
| Specifically, authors need to explain why they did not explore these issues among boys themselves. | We agree with the editor that it is imperative to explore the issues among boys themselves and we have now provided the following reason for focussing on male coaches:  “Given the small scale of the case studies the iALARM project could support, as well as the interest from soccer coaches in better understanding how other soccer coaches experienced and directed their work with boys, this case study only focused on the experiences and perspectives of coaches.” Please see the added sentence from line 179-182 |
| Given soccer is a tough, physical sport, does it not reinforce hegemonic masculinity? | We thank the editor for this comment. To address this comment, we have added the following paragraph from line 110-115:  Indeed, as soccer is a tough, physical sport, it possesses the potential to reinforce some of the more damaging elements of local hegemonic masculinity. Yet, while strength and toughness are traits that are associated with local hegemonic masculine norms, it is not a straight or inevitable line from a more general characteristic (like toughness) to a specific underlying and toxic form of masculinity (27). It is critical to consider exactly how strength or toughness are imagined and operationalized in a local gender order to assess their significance.  Furthermore, while soccer players are expected to demonstrate physical strength and toughness, there is also an awareness that for them to be successful in their soccer careers, they need to have good character, self-control, be able to restrain themselves, and be disciplined (28). As such, youth sport programmes, including soccer clubs, are known for teaching, and emphasizing discipline and etiquette to players including being on time for practice, respect, compassion and caring (29). |
| Authors need to present their understanding of concepts (e.g., gender socialization). | We are thankful to the editor for this suggestion. We have now added the following two paragraphs in the Introduction (line 74-79) to explain our understanding of gender socialization as a concept and how we use it in the paper:  Scholars argue that such learning occurs through gender socialization, a process of learning the culturally and socially ascribed behaviours, positions and ideals seen to be suitable for individual people based on their socially assigned gender (17). Gender socialization occurs through inter-personal relationships and interactions with one’s social milieu, including socializing agents like peers and parents, the setting and circumstances an individual lives in, and the social institutions in which they live such as family, schools, media, and churches (18-20).  In many communities in South Africa, studies have shown that boys are socialised to have dominance and control over girls, including in sexual and romantic relationships (21). Moreover, boys are socialised to perceive themselves to be stronger than girls, take up tasks that are seen as hard or challenging, and to have freedom to experiment with heterosexual sex (21). Boys are also often given more social freedom to move outside the home and partake in leisure activities and are rarely expected to take on household chores (11, 21).  In this paper, gender socialization is understood as a process whereby a social agent (e.g., soccer coach) helps to socialize an individual (e.g., adolescent boy) into a local gender order (i.e. how gender is arranged in a particular place and time) (22), thereby helping to train them into a gendered world. Gender socialization is a process that is independent of any specific gender norms. Boys can be socialized into gender-equitable norms or gender-inequitable norms (or some combination of both). Gender socialization in soccer clubs thus refers to all the ways in which participating in a soccer club might socialize adolescent boys into a particular gender order, not only by explicitly promoting certain gender beliefs but also by showing boys how to relate to each other as boys and what kinds of practices and norms are expected of boys and men in their society. |
| Finally, consider the WHO definition of a gender transformative approach i.e., one ‘that addresses the causes of gender-based health inequities through approaches that challenge and redress harmful and unequal gender norms, roles and power relations that privilege men over women’. | We thank the editor for this suggestion. We have incorporated the WHO definition into the introduction section of the paper. Please see from line 56-59. |
|  |  |
| **Reviewer 1** | **Response from authors** |
|  |  |
| Thank you for the opportunity to review this manuscript. The manuscript describes findings from a qualitative study which aimed to explore how gender socialization of adolescent boys works in soccer clubs, and whether there are opportunities for having gender transformative elements in that socialization. I think this article has the potential to make an important contribution to the literature, but it would benefit from further revision. Please see my comments below: | Thank you. |
| - The authors state several times that this study aims to explore how soccer clubs shape the gender socialization of adolescent boys. The authors then go on to claim that they achieved this aim. However, I’m not fully convinced. I think the manuscript would greatly improve if the authors first define what they mean by gender socialization and then provide more evidence to back up their claim that their findings demonstrate how soccer clubs shape gender socialization of adolescent boys. A lot of the examples in the results section that are described as socialization do not appear to specifically challenge hegemonic masculine norms or promote gender equitable norms. The findings focus much more on how the coaches address challenges related to the boys’ social and economic situation, which in my mind, is not necessarily linked to gender norms. | We thank the reviewer for this comment. In the introduction section (line 74-95) we have now provided our understanding of what gender socialization is as a concept and how it happens. Thereafter in the Results section we have presented more evidence to demonstrate how soccer coaches engaged in gender work with the adolescent boys, and clearly argue that such gender work could either reinforce or challenge conservating gender norms, attitudes, and masculinities. |
| - I appreciate that the authors call for future research to understand these research questions from the boys’ perspective, but I think the fact that the authors did not interview boys for this project should be listed as a limitation. Having the boys’ perspective on their own experience of how the soccer club may/may not shape their gender socialization is critical to our understanding of this process. | We are grateful to the reviewer for raising this point. We have now provided a reason for only focusing on soccer coaches. Please see the added sentence from line 179-182 |
| I think a rationale for why the authors only focused on the soccer coaches is also necessary. | As now clarified in the manuscript, several soccer coaches were members of the iALARM Task Team and had shared their experiences and challenges as coaches of soccer clubs in low-resources communities during the iALARM Task Team meetings. At first, we had informal conversations with them to hear more about their experiences and challenges they were facing in coaching adolescent boys in their clubs. The interest to conduct in-depth interviews resulted from these conversations and the fact that the coaches wanted to know better how to work with the adolescent boys and support them. Furthermore, resources prevented us from expanding this project to include boys. We however agree with the reviewer that there is need to explore the perspectives of the boys in soccer clubs. We thus plan to do this research with boys in soccer clubs as the next step. |
| - In the discussion section (page 24, lines 624-629), the authors state that “our findings show how the coaches’ interventions happened through role modelling, mentoring, educating and supporting (emotionally and socially) the boys to alter their journeys; they challenged boys to critically reflect on risky behaviors and practices, approach life in a more progressive way (e.g., work hard at school and be respectful towards women) …” After reading the results section, I was surprised by this claim because I did not see much of this reflected in the results. More evidence needs to be provided about how the coaches served as role models (and what behaviors or attitudes they role modelled) and how they taught boys to be respectful towards women. I did not see evidence of this in the results section. | We are grateful to the reviewer for this comment. We have now presented more evidence in the findings section showing (see the sub-theme on Coaches as Social Fathers from line 471-521) how the coaches intervened in the lives of the boys through educating them about the dangers of using drugs and engaging in criminal activity and engaging in gender work with the boys, and positioning themselves as role models to the boys through exhibiting appropriate behaviours (e.g., not drinking alcohol) in the presence of the boys. |
| - The authors mention the iALARM Task Team in the abstract, but there is no explanation of what this is. We are only introduced to this term at the end of the introduction. A brief description of what this is should be included in the abstract to help orient the readers. | We have removed reference to having used minutes of the iALARM Task Team meeting as data in this study. Reference to the minutes has been removed both the abstract and manuscript. |
| The authors use the term “gender” in several ways throughout the introduction that needs more attention to make sure the correct meaning is being conveyed. For example, on page 3, line 76, the authors use the term “To transform gender.” I think here the authors mean to challenge harmful/inequitable gender norms. On lines 100-101, the authors state: “There is a belief that the social fathering role represents a real opportunity to promote more positive gender identity.” Again, I do not think this is the right term here. Gender identity refers to the personal sense of one’s own gender (whether they identify as male, female or other). I do not think the authors intended to refer to this, and I’m not sure what a “positive gender identity” is. Instead of gender identify, did the authors mean to say equitable gender norms? | We thank the reviewer for these suggestions. We concur with the reviewer’s view on these concepts and have now changed them accordingly. |
| I also think it would be useful for the authors to unpack the idea of social fathering more. How does it promote positive gender identity- again, do you mean equitable gender norms? I imagine as you mentioned above, the impact of social fathering on boys’ endorsement of equitable gender norms might not necessarily be positive- it depends on the social father’s views. | Thanks to the reviewer for this suggestion. We have now unpacked our understanding and usage of the term ‘social fathering’. We have added the following paragraph in the Introduction (see from line 135-143:  The literature on social fathering suggests that social fathers are those that fill a gap left by an absent (or insufficiently present) father figure in the family. They work to play a fathering role in the lives of these children (30). The concept of social fathering applies whether a child has a biological father or other father figure at home, and the role is often played by men in local social institutions such as the church, school or soccer club. In this article, we use the term as a way of talking about the ways male coaches who are not biological fathers can play both a fathering and gendering role. It is critical to note, however, that the idea of social fathering is not unique to soccer clubs; rather it is part of the general way of thinking about more distributed forms of fatherhood in South Africa (31). |
| Context of study: A little more information about what the iALARM task team does would be useful here and how the soccer clubs fit into this parent study. | We have now provided more information about the iALARM Task Team and showed how soccer clubs were a part of the NGOs in the Task Team. |
| You say you analyzed iALARM meeting minutes, but it remains unclear how are those relevant to your research questions. Who is included in these meetings? What is discussed? | We have removed reference to the iALARM meeting minutes as the minutes had contributed very little to the data analysed in this paper. As such, we have removed the quote that we had extracted from the iALARM Task Team meetings. |
| As noted earlier, I think a rationale needs to be provided for why you didn’t also explore the perspectives of the boys themselves. | We trust that the response we provided above addresses this comment. |
| Again, a rationale for reviewing the iALARM meeting minutes would be helpful. Why are these relevant to your research question? What information were you trying to learn from these meetings? | Thanks to the reviewer, as indicated above, we have removed reference to the iALARM meeting minutes and no longer use the minutes as data in this paper. |
| Where were the interviews conducted? | We have now added a sentence in the methods section explaining that the IDIs were either conducted in-person in a community hall or telephonically by the 1st author. Please see from line 236-237. |
| As noted previously, the authors need to provide more evidence of the gendered socialization within the soccer clubs. | As stated above, we have now presented more evidence of gender socialization within soccer clubs. Please see under the sub-theme Coaches as Social fathers’ from line 471-521. |
| The quotes shared for the section on complexities around the social fathering role do not clearly illustrate how playing this role is “not easy” or that “there are complexities associated with playing it.” I suggest you add more quotes to better articulate what you mean by this. | We are grateful to the reviewer for this suggestion. First, we have revised and reorganised our Findings section and the themes and sub-themes. In the revised manuscript, the complexities around the social fathering role are now presented under the sub-theme ‘Social fathering and the family at home’. As suggested by the reviewer we have added more quotes under this sub-theme to clearly show how playing this role is not easy. See from line 545-619. |
| More quotes are needed to illustrate coaches frustrations with navigating tenuous dynamics with parents. | Thanks to the reviewer for this suggestion. We have added 2 more quotes from 596-615 to illustrate the coaches’ frustrations with navigating tenuous dynamics with parents. |
|  |  |
| **Reviewer 2** |  |
|  |  |
| Why did the authors conduct 11 interviews? | We thank the reviewer for this comment. To respond to this comment, we have added the following paragraph from Please see from line 238-241.  We stopped conducting the interviews at interview 11 for two reasons. First, there were a limited number of soccer coaches who trained adolescent boys in both Gugulethu and Mthatha. Second, when we reached interview 9 & 10, we felt that we had reached data saturation, as similar themes were being elicited from the interviews to answer the exploratory areas of interest. |
| How did the authors recruit the participants for actual fieldwork? | Under ‘Sampling and participants’ (from line 215-225) we have now provided further description of the participants recruitment process. |
| Did the participants that attend interview informed about the research??/ | Thanks to the reviewer for this question. All participants were informed about the research. The first author first explained the purpose of the research and those interested to participate were asked to give informed consent prior to the interview. |
| The authors must correct error on line 422, they wrote “a” instead of “are” | Thanks for the reviewer for pointing out this error, it has been corrected. |
| Another error, “instead of couching” authors wrote” couches” | We thank the review for pointing out this typographical error. It has been corrected. |
| May I request the authors to account on how they applied reflexivity to enhance the quality of the results? | We are thankful to the reviewer for this suggestion. From line 815-829 we have added the following paragraph as our account on how we applied reflexivity in this study to enhance the quality of our data and findings.  ***Reflexivity***  The authors are researchers, who are situated within both academia and practice. Two of the authors were born and had lived in the two settings of this study, as adolescent boys and amateur soccer players (YS and MM) and were able to provide insider perspective on this topic. Their prior experiences in these settings may potentially bias their views on the analysis of the data. Other authors (CC, NL & ML) are White academics who have lived for many years in the Western Cape province where they conducted extensive health systems research, community development work, health promotion work, and gender transformative interventions with boys and men in Black poor communities in the Western Cape province, including Gugulethu. As such, while they have extensive knowledge on this topic and highly skilled in qualitative data analysis and interpretation, it is possible that their personal experiences, identities, and social positions may have shaped their interests in this work and therefore how they interpreted the data. Notwithstanding, the discussion and refining of interpretations across the author team ensured that the findings and interpretations reported here were grounded in the data collected in the study. |
| The first theme and sub theme are specifically about coaches’ perceptions of adolescent boys’ problems / social problems boys are facing. - Is TMC a social problem? Or behavioural aspects of adolescents who attend this rite of passage to manhood present social problems? | We thank the reviewer for this query and suggestion on how we can frame TMC in the context of this sentence. Please see the revision from line 327-333. |
| - The authors seem to think all the boys that attended TMC only start dagga, alcohol etc when they were in initiation school, however research shows that adolescent start these as early as 14 years or even younger. | We agree with the reviewer that some adolescent boys start using substances at an early age. However, in line with the coaches’ views in our study, some qualitative studies from traditional circumcising communities in South Africa do suggest that some adolescent boys first experience with substances during their time in the initiation school, with them being introduced to these substances by the amakhankatha (traditional guardians) who were looking after them (see Prusente et al. 2019).  Prusente, S., Khuzwayo, N., & Sikweyiya, Y. (2019). Exploring factors influencing integration of traditional and medical male circumcision methods at Ingquza Hill Local Municipality, Eastern Cape: A socio-ecological perspective. African Journal of Primary Health Care & Family Medicine, 11(1), 1-11. |
| - What are the ages of the adolescents that are being coached. TMC in Eastern Cape and Cape Town provinces is only allowed in boys from 18 years and older -if the coaches can prevent such behaviours until initiation; I would also recommend soccer avenues. line 497, some coaches said they are working with boys/men from 8-17 years this is before they go to initiation, do they also have boy who went before 18 years? | According to the coaches, the ages of the adolescent boys they coached in their clubs ranged between the ages of 08 and 19 years, although in some teams there were older players.  The reviewer is correct that by law in South Africa only boys who have reached the age of 18 can undergo TMC. However, while it is unlawful, it does happen that boys younger than 18 undergo TMC. However, the coaches did not necessarily mention this in the interviews. |
| - This theme/sub theme doesn’t clearly articulate or describe the research questions from line 117-120  - Maybe the authors may consider defining what are social problems in their study first, before they attempt this theme/ subtheme. Line 485: Complexities around the social fathering role | We thank the reviewer for raising this query. We agree that the theme on ‘Complexities around the social fathering role’ does not appear to describe the research questions. In response to the reviewers’ comments, we have reorganised our findings section for a better flow and coherence. We now have two main these, the first being a revised theme on ‘Coaches’ perceptions of adolescent boys problems’. Under this theme, we now first provide a definition of the social problems as follows:  The coaches identified three main areas of problems and underlying dynamics associated with these problems. These are social and behavioral problems related to substance abuse and gangsterism; emotional problems related to the family dynamics and home stressors; and class differences related to socio-economic background. See from line 284-287. |
| LINE 497- 500 and 507-514: The excerpts presented are describing complexities of being a soccer coach. I am saying this because authors interviewed coaches. Coaches are biological fathers first; biological fathers are expected to support their boys. Now when these biological fathers become coaches, they are expected to fill in all these responsibilities including social fathering. | We thank the reviewer for this comment. We wish to clarify that the coaches we interviewed in this study were not the biological fathers of the boys in their clubs, rather, upon seeing the challenges faced by the boys, they took on the responsibility of playing a social fathering role. To make this clearer, we have reorganised our Results sections. We now first present a theme on Coaches’ perceptions of adolescent boys’ problem’s and then a second one on ‘Social fathering & gender socialization. Under the second theme, we have a sub-theme on ‘Social fathering and the family at home’ where show that the complexities arose mainly because the coaches were not biological fathers of the boys. Because of this, the coaches couldn’t fully involve themselves in the lives of the boys and indeed some coaches talked about having tensions with the parents of the boys because they were perceived by the parents to be overstepping the boundary of being soccer coaches. |
|  |  |
| **Reviewer #3** |  |
|  |  |
| This is an important paper which seeks to assess how gender socialization of adolescent boys works in soccer clubs, and whether there are opportunities for having gender transformative elements in that socialization. The authors need to deal with few things to get the paper to a more solid place. | We thank the reviewer for the positive appraisal of our study. We trust that the revisions we have made in the manuscript have further strengthened the manuscript. |
| 1. The paper uses concepts such as "gender socialization" of adolescent boys and "gender transformative" without clearly defining what they mean. Gender Socialization is the process of educating and instructing males and females as to the norms, behaviors, values, and beliefs of group membership as men or women. Is this what the coaches were doing? What are the acceptable ways of being a boy in the study area-- to be fearless, competitive, heterosexual etc? I am curious to know what gender transformative socialization means in this context? It will be key to clarify what definitions of these concepts that the authors now apply to the coaches' work. | We are grateful to the reviewer for this insightful comment. To address this comment, we have now presented in the Introduction the definitions of the key concepts we are using in this paper. We have added the definitions of gender socialization, gender-transformative approach and social fathering and our understanding of these concepts and how we use them in the paper. |
| Of course, soccer can be a platform for gender socialization, but is also a very competitive sports that builds out tendencies for striving, endurance, not showing pain, violence, and extreme hardiness in boys etc. Were these part of the virtues the coaches sought to instil in the boys? | We concur with the reviewer that soccer is a very competitive sport that builds out tendencies as described by the reviewer. To address this point, we have added the following paragraph in the Introduction:  Indeed, as soccer is a tough, physical sport, it possesses the potential to reinforce some of the more damaging elements of local hegemonic masculinity. Yet, while strength and toughness are traits that are associated with local hegemonic masculine norms, it is not a straight or inevitable line from a more general characteristic (like toughness) to a specific underlying and toxic form of masculinity (27). It is critical to consider exactly how strength or toughness are imagined and operationalized in a local gender order to assess their significance.  Furthermore, while soccer players are expected to demonstrate physical strength and toughness, there is also an awareness that for them to be successful in their soccer careers, they need to have good character, self-control, be able to restrain themselves, and be disciplined (28). As such, youth sport programmes, including soccer clubs, are known for teaching, and emphasizing discipline and etiquette to players including being on time for practice, respect, compassion and caring (29). |
| The focus of the coaches' engagement with the boys were on sexual and reproductive health, alcohol and drugs and involvement in criminal gangs. And in some cases, supporting the boys to think about their life journeys and change their risky behavior and practices. But gender socialization also goes well beyond these to include traits and practices that are also harmful to boys. | We agree with the reviewer that gender socialization goes well beyond engaging with someone on issues such as SRH, substance use and gangsterisms. In the Results, we have now presented more evidence to show how the coaches engaged in gender work with the boys, with some coaches being more deliberate on supporting the boys to construct alternative masculinities. In our interpretation of these argument that this gender work could go either way, in that the coaches may reinforce traditional norms or masculinities or support the boys to construct gender-equitable masculinities. To argue this point, we have added the following paragraph in the Discussion section:  In our study, the coaches emphasized to the boys the significance of self-respect, respect for women and elders, discipline, and positive contribution to society. This suggests that they aimed to socialize the boys to construct alternative forms of masculinities which embody these values and principles. Yet, this does not necessarily mean the masculinities they were producing were gender-transformative, as boys and men may embody traditional values of respect, including respect for girls and women, yet not view girls and women as equal to them. Moreover, while the alternative masculinities the coaches were working towards sought to denounce violence and unhealthy practices, we contend that there are no guarantees that these alternative masculinities would be gender-equitable and transformative. Rather the coaches may reinforce traditional masculinities which are health promoting (i.e. avoid engaging in violence, gangsterism and use of illicit drugs). |
| 2. The authors need to build a paragraph or two on the context of gender, particularly in relation to adolescence in SA or in the area of the study. The literature reviews lays out the debates and research on soccer and gender, but it is silent on the issue of gender socialization and gender and adolescence in the study area. Addressing this gap will add clarity to why this study is important. | Thanks to the reviewer for this suggestion. To provide context of gender and adolescence in sub-saharan Africa, we have added the following paragraph from line 81-86:  In many communities in South Africa, studies have shown that boys are socialised to have dominance and control over girls, including in sexual and romantic relationships (21). Moreover, boys are socialised to perceive themselves to be stronger than girls, take up tasks that are seen as hard or challenging, and to have freedom to experiment with heterosexual sex (21). Boys are also often given more social freedom to move outside the home and partake in leisure activities and are rarely expected to take on household chores (11, 21). |
| 3. The paper talks about the coaches' beliefs that traditional male circumcision (TMC) as a key mechanism for socializing boys longer performs its intended socializing role. But what is it that TMC did in the past that it is was able to "properly" socialize boys in the past? | We thank the reviewer for this comment. To address the comment, we have added the following paragraph, from line 725-734, in the Discussion section:  Research suggests that in the past TMC succeeded in its intended role of socializing young boys into local norms of respectful, responsible men because of certain elements it possessed (44). These included the active involvement of elderly men who closely monitored the initiation process and frequently visited the initiation schools to ensure that only socially acceptable behavioral practices and values were taught to the initiates (44). Furthermore, studies suggest that in the past there was careful selection of amakhankatha [traditional nurses] by the family of the initiate, with only elderly men who were of a high social standing and ‘lived exemplary lives’ taking the role of being a traditional nurse (44). Such men were able to teach and transfer to the initiates moral values such as self-respect and respect for others and social responsibility (44, 45). |
| 4: I think a more fundamental issue is the lack of a critical appraisal of the kind of gender socialization that these boys were receiving, that whether on one hand they were re-inscribing traditional notions of masculinity/gender that could also harm the boys, which soccer is globally known for. | We are thankful for the reviewer for this important comment. We have now critically appraised the kind of gender-socialisation they boys were receiving in the clubs. In addressing this comment, we have added the following paragraph, from line 767-773, in the Discussion section:  In our study, the coaches emphasized to the boys the significance of self-respect, respect for women and elders, discipline, and positive contribution to society. This suggests that they aimed to socialize the boys to construct alternative forms of masculinities which embody these values and principles. Yet, this does not necessarily mean the masculinities they were producing were gender-transformative, as boys and men may embody traditional values of respect, including respect for girls and women, yet not view girls and women as equal to them. |
| What was the ultimate boy-product that these coaches wanted to see. This needs to come out clearly in the study. What additional social(ization)work is needed with these boys from the perspective of the coaches? This is not clear too. | Thanks again to the reviewer for this insightful comment. To address the comment, In the Discussion we have added the following paragraph (from line 773-777), linking it with the one above:  Moreover, while the alternative masculinities the coaches were working towards sought to denounce violence and unhealthy practices, we contend that there are no guarantees that these alternative masculinities would be gender-equitable and transformative. Rather the coaches may reinforce traditional masculinities which are health promoting (i.e. avoid engaging in violence, gangsterism and use of illicit drugs). |
|  |  |

We hope that this resubmission meets with the requirements of PloS One.

Kind regards,

Yandisa Sikweyiya
