# Supplementary material for: Soccer clubs as avenues for gender transformative socialization of adolescent boys in Cape Town and Mthatha, South Africa: A qualitative study
Source: PLoS One. 2023 Feb 2;18(2):e0280932. doi: 10.1371/journal.pone.0280932 (PMC9894412; doi:10.1371/journal.pone.0280932)
Supplement: Attachment — Submitted filename: Response to Reviewers.docx [file pone.0280932.s003.docx]

Dear Prof Webster Mavhu,

Title: The potential of soccer clubs as avenues for gender transformative socialization of adolescent boys in Cape Town and Mthatha, South Africa: findings from a qualitative study’.

A sincere thank you to reviewer 3 for giving us a further opportunity to have our paper revised and resubmitted to PloS One. Thank you also for the detailed suggestions provided.

We have carefully considered reviewer 3’s further comments to ensure that we adequately address them. In the table below we have summarised the revisions that we have made in response to reviewer 3’s comments.

All the comments are listed and responses from the authors are given accordingly.

| **Editor** | **Response from authors** |
| --- | --- |
|  |  |
| **Reviewer #3** |  |
|  |  |
| The authors have done their best to respond to the comments. But they could do better through critical engagement with their data and my earlier comments. I am reviewer 3 and I feel my earlier comments were parried rather than addressed in any solid way. | Thanks to the reviewer for the positive feedback. We have considered the reviewer’s comments and revised the paper in line with the reviewer’s comments. |
| What rings very clear to me appears to be that the coaches are interested in supporting and molding out a soccer masculinity that will make their coaching careers successful. It may or may not be any conscious effort to mold a new gender equitable male youth in South Africa. | We are grateful to the reviewer for this insight and interpretation of our findings. We agree with the reviewer’s interpretation. We have now revised the discussion section and added the following paragraph on page 34, lines 762-768.    ‘We argue that through such gender work, the coaches were intending to shape and reinforce a positive soccer masculinity (49) among the boys, one that will make both their coaching careers and boys’ soccer careers successful. However, in engaging in this gender work, it is unclear whether the coaches had made a conscious effort to shape a new gender equitable male youth. This warrants further exploration through targeted in-depth research on the coaches’ role and motivations for engaging adolescent boys in sports.’ |
| In my previous review, I asked : What is the ultimate boy-product that these coaches wanted to see? Hard as this question appears, it lies at the heart of transformative gender work and partly defines your work contribution to the field. It's difficult to appreciate the work of the coaches, if the authors did not ask them to articulate a clear and shared vision of this new type of male youth their social fathering intends to produce? Or if the coaches themselves had no vision of what the new young man they want to create will look like in real life. Were they just hoping to produce good and committed soccer youth? Several successful sportsmen are also known for abusive behaviour towards children, women and other men! So what were they looking to create ultimately? | We thank the reviewer for this question. We agree that this is an important question to address in a study concerned with transformative gender work with boys. However, on page 7, lines 151-159 we have described the topics/questions that were asked to the coaches interviewed in this study. Unfortunately, the question about the ‘ultimate boy-product the coaches wanted to see’ was not directly asked to them. As such, our data do not allow us to adequately address this question in this paper. We have now acknowledged this as a limitation of our study by adding the following paragraph on page 36, lines 807-812.  ‘The scope of our study is limited on the question relating to the ultimate boy-product the coaches wanted to see when they engaged in the social fathering role with the boys in their clubs. As such, our data do not allow us to adequately address this question in this paper. Future studies exploring the social fathering role played by soccer coaches and how it may work to socialize boys into a gender order in a particular setting would do well to explore this question. Exploring this question is critical as it lies at the heart of transformative gender work with adolescent boys.’ |
| The authors still appear to conflate mentorship and social fathering. They appear to proceed from the argument that fathering, and parenting are inadequate and poor, and that 's the problem of these young men. But the young men described in the coaches' narratives appear to be a troubled lot, dealing with the structural fallout of persistent and overwhelmingly violent apartheid system and colonial history. Societal values have moved forward and fast, creating a vast army of youth with little preparation and asset to tap into emerging opportunities or compete well in the new society. These are youth left behind in a country with the world's worst GINI coefficients. It is not poor fathering. This context is key to understand before we blame it on fathering and seek a solution that merely tackles symptoms rather than causes. Still, it appears to me that the football platform appears to offer some form of useful entry point to draw young people away from some vices, but real re-socialization into a new norms of gender requires key structural changes that incorporate a focus beyond sports. Work is also needed at the household level where the youth return to, but where the coaches have little leeway to carry that work through. And, it is also really not their work and mission. Efforts and initiatives that seek to identify entry points for gender transformative work but focus little on the household and other critical settings that nest youth will have limited impacts. | We thank the reviewer for this comment. Indeed, we agree on the historical and structural roots of the problems and challenges faced by the youths as described by the coaches, and how these shaped the lives of the youths described in this paper and would not want our insights to be read as attributing personalized blame on families and men. In addressing the comment, we have added the following paragraph in the Discussion section (see page 35, lines 774-785).  ‘Our findings also suggest that soccer clubs offer a useful entry point to draw adolescent boys away from social vices (e.g., using illicit drugs and involvement in gangsterism), yet we contend that to succeed in re-socializing adolescent boys into health-promoting norms of gender, there is a need for structural changes that incorporate a focus beyond sports. Specifically, in South Africa, there is an urgent need for structural interventions aimed at improving the livelihoods of working-class people in resource-poor communities. Most Black South African communities are characterized by high levels of poverty, unemployment, and dysfunctional families (51). Thus, our finding on absent or uninvolved fathering in the families of some boys in soccer clubs should be understood in the context of economic disenfranchisement of Black people which has its roots in colonial and apartheid systems (52), and the continued socio-economic, socio-political, and structural forces in democratic South Africa that continue to adversely affect and shape the lives of adolescent boys in poor Black communities.’ |
| There is still little engagement with the concept of transformative gender work. The definition used for this is taken for granted and not problematized. | Thank you. We agree that transformative gender work is complex to unpack and that this paper is limited in its scope. We hope that the revisions made in this draft indicate that we now tread more cautiously about making claims of the transformative nature of the gender work done by the coaches. |
| The authors' response to the question of how TMC socialized boys is mere romanticism. | We thank the reviewer for this comment. We have now revised the discussion section and added the following paragraph on page 33, lines 718-728.  ‘Historical literature from South Africa suggests that in the past TMC succeeded in its intended role of socializing young boys into local norms of respectful, responsible men because of certain elements it possessed (43, 44). Hellman (45) has noted that, in the past, TMC in Black rural communities was more of a group process that enabled a sense of an age cohort―with initiates positively influencing each other, yet in more recent times, TMC has largely become an individual process. Secondly, scholars like Wilson and Mafeje (46) observed in Langa township, Cape Town, that in contemporary times, the emphasis is put on the clinical operation (circumcision), with very little focus on the educational aspect of the custom (see also 47). Moreover, Wilson and Mafeje (46) observed that in more recent times the period of seclusion for initiates is shorter, this limiting the time for the initiates to be educated on particular attitudes, moral values such as self-respect and respect for others, and social responsibility.’ |
| The study also needs to decolonize itself. It falls into the traps of assuming that gender values are an ideal. | Thank you. We are unsure how to address this issue. We hope that the revisions made in this draft reflects a more cautious approach to socio political context in which this study takes place. |
|  |  |

We hope that this resubmission meets the requirements of PloS One.

Kind regards,

Yandisa Sikweyiya
