# Supplementary material for: Soccer clubs as avenues for gender transformative socialization of adolescent boys in Cape Town and Mthatha, South Africa: A qualitative study
Source: PLoS One. 2023 Feb 2;18(2):e0280932. doi: 10.1371/journal.pone.0280932 (PMC9894412; doi:10.1371/journal.pone.0280932)
Supplement: Attachment — Submitted filename: Manuscript - Potential of soccer GT socialisation_wm.docx [file pone.0280932.s004.docx]

**The potential of soccer clubs as avenues for gender transformative socialization of adolescent boys in Cape Town and Mthatha, South Africa: findings from a qualitative study**

Yandisa Sikweyiya^1.2*^, Natalie Leon^3,4^, Mark N. Lurie^4,5^, Mandla Majola^5^, Christopher J. Colvin^4,5,6^

1. Gender and Health Research Unit, South African Medical Research Council, Pretoria
2. School of Public Health, Faculty of Health Sciences, University of the Witwatersrand, Johannesburg, South Africa.
3. Health Systems Research Unit, South African Medical Research Council, Tygerberg, South Africa
4. Department of Epidemiology, Brown University School of Public Health, Providence RI, USA
5. Division of Social and Behavioural Sciences, School of Public Health and Family Medicine, University of Cape Town, Cape Town, South Africa
6. Department of Public Health Sciences, University of Virginia, Charlottesville, VA, USA

*Yandisa Sikweyiya, Gender and Health Research Unit, South African Medical Research Council, Private Bag X385, Pretoria, 0001, South Africa; Email: [yandisa.sikweyiya@mrc.ac.za](mailto:yandisa.sikweyiya@mrc.ac.za)

# Abstract

# In this paper, we explore the gender socialization of adolescent boys in soccer clubs, and ask whether there are opportunities for integrating gender transformative elements into that socialization. This qualitative study involved 11 in-depth interviews and informal conversations with male soccer coaches from Gugulethu township and Mthatha town in the Western Cape and Eastern Cape provinces of South Africa, respectively. Data were analyzed using a thematic analysis approach. We found that the coaches felt that the adolescent boys in their soccer clubs faced serious social and emotional challenges, with the boys’ poor socio-economic backgrounds and fragmented family structures being major contributors to these challenges. Most coaches also gave themselves the responsibility to try to address some of the challenges faced by their club members. To do this, they employed specific strategies, including creating an alliance with parents and professionals. In the process, the coaches engaged the boys on topics around respect, sexual and reproductive health, and avoiding alcohol, drugs, and involvement in criminal gangs. Some coaches also played a ‘social fathering’ role to club members as a way of helping them to think differently about their lives, redirect risky practices, and reduce the chance for poor health outcomes. These findings highlight the role of soccer clubs and coaches as avenues for health- and equity-promoting gender socialization of adolescent boys.

# Introduction

Globally, gender scholars have underscored the need to engage boys and men in gender-transformative programming to challenge gender inequality (1, 2). A gender-transformative approach is one ‘that addresses the causes of gender-based health inequities through approaches that challenge and redress harmful and unequal gender norms, roles and power relations that privilege men over women’ (3). As further explained by Jewkes and others (4), gender-transformative approaches typically work with groups of boys or men to enable critical reflection about gender roles, norms, and power relations between males and females. Such work is considered vital to support boys and men to construct non-violent masculinities (5, 6), reduce their risky behaviors, and improve their health-seeking practices (7-9). A growing body of literature suggests that gender-transformative programming with boys and men has the potential to support a shift away from harmful gendered norms and practices that could place them and their partners at risk of acquiring HIV and other poor health outcomes (2, 4, 9, 10).

Some gender-transformative interventions implemented in South Africa have been found to be promising or effective in enhancing boys’ and men’s linkage and retention in HIV care (see 7), lessening their involvement in violence victimization and perpetration (11, 12) and improving their sexual and reproductive health (SRH) outcomes (13). Scholars have identified the adolescent phase as a critical time in which to intervene in the development of health, gendered and sexual behaviors (10, 14, 15). This is because, as Blum, Mmari & Moreau (16) contend, adolescence is a time when boys learn most intensively how to construct their masculinities in line with cultural and social expectations of being a man in their settings. Scholars argue that such learning occurs through gender socialization, a process of learning the culturally and socially ascribed behaviors, positions, and ideals seen to be suitable for individual people based on their socially assigned gender (17). Gender socialization occurs through inter-personal relationships and interactions with one’s social milieu, including socializing agents like peers and parents, the setting and circumstances an individual lives in, and the social institutions in which they live such as family, schools, media, and churches (18-20).

In many communities in South Africa, studies have shown that boys are socialized to have dominance and control over girls, including in sexual and romantic relationships (21). Moreover, boys are socialized to perceive themselves to be stronger than girls, take up tasks that are seen as hard or challenging, and have the freedom to experiment with heterosexual sex (21). Boys are also often given more social freedom to move outside the home and partake in leisure activities and are rarely expected to take on household chores (11, 21).

In this paper, gender socialization is understood as a process whereby a social agent (e.g., soccer coach) helps to socialize an individual (e.g., adolescent boy) into a local gender order (i.e. how gender is arranged in a particular place and time) (22), thereby helping to train them into a gendered world. Gender socialization is a process that is independent of any specific gender norms. Boys can be socialized into gender-equitable norms or gender-inequitable norms (or some combination of both). Gender socialization in soccer clubs thus refers to all the ways in which participating in a soccer club might socialize adolescent boys into a particular gender order, not only by explicitly promoting certain gender beliefs but also by showing boys how to relate to each other as boys and what kinds of practices and norms are expected of boys and men in their society.

Some have argued that soccer clubs are ideal vehicles for gender transformative socialization among adolescent boys in South Africa (23, 24). A growing literature suggests that important gender socialization work is indeed happening in soccer clubs (23, 25). However, not all that socialization is gender transformative. Some of it may reinforce inequitable gender norms (e.g. men’s superiority, control, and dominance over women) and dominant or destructive masculinities that emphasize power and force (26). Gender work in soccer clubs could also promote masculinities that value sexual prowess and conquest in heterosexual relationships, practices that are associated with multiple and concurrent sexual partnerships, sexual risk-taking, unprotected sex, and sexual assault on intimate partners (14). Indeed, as soccer is a tough, physical sport, it possesses the potential to reinforce some of the more damaging elements of local hegemonic masculinity. Yet, while strength and toughness are traits that are associated with local hegemonic masculine norms, it is not a straight or inevitable line from a more general characteristic (like toughness) to a specific underlying and toxic form of masculinity (27). It is critical to consider exactly how strength or toughness are imagined and operationalized in a local gender order to assess their significance.

Furthermore, while soccer players are expected to demonstrate physical strength and toughness, there is also an awareness that for them to be successful in their soccer careers, they need to have good character, and self-control, be able to restrain themselves and be disciplined (28). As such, youth sports programmes, including soccer clubs, are known for teaching, and emphasizing discipline and etiquette to players including being on time for practice, respect, compassion, and caring (29). Again, while these characteristics can be woven into either gender-equitable or gender-inequitable masculinities, gender transformative strategies seek to direct these pro-social values into the construction of alternative masculinities which are more gender equitable and reject the use of violence on women and other men (5).

We must understand how gender socialization happens in soccer clubs if we want to take advantage of the potential opportunities soccer clubs offer for a potential pathway to transformative gender work. There are several mechanisms through which gender socialization happens in soccer clubs. For instance, by keeping adolescent boys engaged, enabling them to exercise and be healthy, giving them a sense of achievement, and being part of a larger group, learning about team play, problem solving, practice for improvement, and providing them with a sense of hope for a better future, soccer clubs can profoundly shape the gendered norms and practices of adolescent boys (25).

While a great deal of gender work may happen via the planned activities of the soccer club, the relationship between the players and the coach is another important domain the work of gender socialization happens. These relationships of course take different forms and shapes. Coaches can serve as positive or negative role models, or implement discipline in punitive or positive ways, as shown in Medich et al.’s study (23). When these relationships and processes of disciplining and role-modeling are particularly intensive and evolving, scholars have sometimes framed them through the lens of ‘social fathering’. The literature on social fathering suggests that social fathers are those that fill a gap left by an absent (or insufficiently present) father figure in the family. They work to play a fathering role in the lives of these children (30). The concept of social fathering applies whether a child has a biological father or other father figure at home, and the role is often played by men in local social institutions such as the church, school, or soccer club. In this article, we use the term as a way of talking about the ways male coaches who are not biological fathers can play both a fathering and gendering role. It is critical to note, however, that the idea of social fathering is not unique to soccer clubs; rather it is part of the general way of thinking about more distributed forms of fatherhood in South Africa (31).

The idea that a male soccer coach can or should serve a fathering role for adolescent boys is an important component in the promotion of soccer clubs. However, our understanding of soccer coaches as social fathers is limited. Not much is known about how this works in soccer clubs, as research on social fathering in South Africa has mainly been done with adult men in community-based studies (31). Scholars have recently identified this idea as relevant to soccer clubs (23, 25), but there is little work referring to the coaches themselves and their experiences in the social fathering role.

In this paper, we explore how gender socialization works in soccer clubs, and whether there are opportunities for integrating gender transformative elements into that socialization. Specifically, we explore the dynamics between coaches and boys, from the coaches’ perspective, to learn more about how the coaches are—or could be—playing a mentoring role in supporting the gender socialization of adolescent boys. Moreover, we explore the extent to which this socialization involves a social fathering role (as opposed to, or in addition to other mechanisms for gender socialization). The paper is framed around the following questions: 1) How does gender socialization work in soccer clubs? 2) Are there opportunities for such socialization to be more gender transformative? 3) How does social fathering work to support and achieve gender socialization in soccer clubs?

## **Context of the study**

This study was part of a suite of case studies conducted under the larger project called iALARM (Using Information to Align Services and Link and Retain Men in the HIV Cascade), a 5-year, NIH-funded research project on men and HIV based at the University of Cape Town, South Africa and partnering with Brown University, USA (R01-MH106600). As described by Colvin and colleagues (32), iALARM’s central intervention was the creation of a ‘Linkage and Retention in Care Task Team’, also referred to as the iALARM Task Team in the community of Gugulethu in Cape Town. The Task Team was comprised of various stakeholders including representatives of the local clinic, staff and managers from other health facilities, officials from local government sub-district offices, and representatives of several local non-governmental organizations (NGOs). The NGOs involved in the Task Team included ones focusing on child and adolescent wellness, youth sports (including soccer clubs), parenting and community health, food security, and disability (32). The initial idea for this study emerged out of discussions among iALARM Task Team members about the role of soccer clubs as potential sites for gender transformative intervention. Some of the soccer coaches interviewed in this study were members of the iALARM Task Team, while those from Mthatha were not. This study is one of several sub-studies within the iALARM project that examined community- and health system-based strategies for mobilizing men and boys around HIV and gender equity. Given the small scale of the case studies the iALARM project could support, as well as the interest from soccer coaches in better understanding how other soccer coaches experienced and directed their work with boys, this case study only focused on the experiences and perspectives of coaches.

# **Material and Methods**

## **Study design**

This was an exploratory qualitative study that gathered data through in-depth interviews (IDIs) and informal conversations with soccer coaches. The study design was guided by our interest in developing an in-depth appreciation, from the perspectives of the adult male soccer coaches, on the following issues: their coaching background; the background of the adolescent boys in their clubs; the main issues that come up in coaching; their lived experiences about balancing training soccer and teaching lessons within their clubs that could apply to the boys’ lives off the field; addressing the social well-being of boys; dealing with boys’ experiences of challenging and disadvantaged home backgrounds, school and community environments; interpersonal and emotional issues boys encountered; boys’ dating and sexual relationships; and the strategies coaches were implementing in socializing adolescent boys in their clubs. The interview guide for the IDIs was framed around these questions (see S1 file).

## **Setting**

In Cape Town, Western Cape province, data collection was conducted with soccer coaches who resided in the township of Gugulethu—a peri-urban township with approximately 100,000 people (33). South Africa’s high unemployment rate of 29.1% (34), and very high youth unemployment are long-standing problems. Gugulethu is characterized by high levels of poverty and high rates of interpersonal violence including homicide, gangsterism, and substance abuse. In Mthatha, Eastern Cape province, data collection was conducted with coaches who lived in townships and villages in and around Mthatha—a small town in the OR Tambo District. The province has the highest unemployment rate in South Africa at 37.4% (34), and Mthatha is characterized by limited economic resources, a high crime rate, harmful use of alcohol, and the use of illicit drugs among the youth (35, 36). The area is also characterized by low levels of school completion and particularly high school dropout before the age of 18, though youth who dropped out earlier in their adolescence regularly re-enter school some years later (37).

## **Sampling and participants**

Eleven Black African male coaches were purposively selected to participate in IDIs. First, during the iALARM Task Team meetings, we approached soccer coaches who were present, informed them of our research and its purpose, and invited them to participate in one-on-one interviews. All coaches who were approached agreed to participate were interviewed, and thereafter offered to share names and contact details of other coaches who were part of their social networks in Gugulethu. We then called the suggested coaches and those whose soccer clubs were training adolescent boys were invited to participate in the study and were also interviewed. To enable a rural-urban comparison, the first author used his network in Mthatha to identify coaches who had soccer clubs that trained adolescent boys in Mthatha townships and villages. He requested a soccer coach he knew to introduce him to other soccer coaches in Mthatha, and those who confirmed that they were training adolescent boys were invited to participate in the interviews. In total, five coaches from Gugulethu and six coaches from Mthatha were interviewed. The coaches were between the ages of 30-50 years, all had completed grade 12, four had a tertiary qualification, and six had formal soccer coaching certificates of different levels. The coaches’ experience in training adolescent boys ranged between 3 and 23 years. Coaches reportedly spent approximately five days a week in this role during the soccer season.

## **Data collection procedures**

The data were collected through in-depth interviews (IDIs) and informal conversations with soccer coaches residing in Gugulethu, Cape Town, and townships and villages in and around Mthatha in the Eastern Cape province of South Africa. IDIs were conducted either in-person in a community hall or telephonically in isiXhosa ̶ the dominant spoken language in Gugulethu and Mthatha.

We stopped conducting the interviews at interview 11 for two reasons. First, there were a limited number of soccer coaches who trained adolescent boys in both Gugulethu and Mthatha. Second, when we reached interviews 9 and 10, we felt that we had reached data saturation, as similar themes were being elicited from the interviews to answer the exploratory areas of interest. The digitally recorded interviews were conducted by the first author who is an experienced qualitative social science researcher, male, multi-lingual, and a first language isiXhosa speaker who originates from Mthatha where the rural case study setting is located. The audio-recorded interviews were then transcribed verbatim and simultaneously translated into English by the first author in preparation for analysis.

## **Data analysis**

Data were analyzed inductively using a thematic analysis approach (38), guided by the research question and interview categories of interest. Yet, there were deductive elements to the analysis (39). The first (YS) and last author (CC) were both involved in various stages of data analysis. We first read and re-read the transcripts individually to familiarize ourselves with the content of the transcripts. Next, we established some general codes which somewhat resembled the questions in the interview guide. Text which seemed to fit together was then grouped under distinct codes. Next, the first author examined the data and identified several further ‘open’ codes, which provided information beyond the initial interview questions, which he discussed with the last author. Through an iterative process, open codes were discussed and those considered to be alike were grouped under clearly distinct themes. Afterward, we assessed the patterns and links between the themes and interpreted what we saw emerging.

## **Ethical considerations**

This study was approved by the University of Cape Town Faculty of Health Science’s Human Research Ethics Committee (# XXX). Before conducting the IDIs, the first author reviewed the participant information and consent form with all participants (coaches). The discussion included the rights of participants in the study and the risks and benefits of participating in the study. Verbal informed consent was obtained from all participants before they were interviewed. We have replaced the names of study participants with pseudonyms to protect the identity of the participants. No financial incentive was given to participants for their participation in the study, and this was communicated to the participants before conducting the interviews.

# Results

We have organized our discussion of the results into two main sections. The first section provides an account of how coaches perceived the problems adolescent boys were facing and the underlying reasons for these problems. The challenges included social and behavioral problems, emotional stresses, and class tensions within their communities. The second section explores how coaches, by playing a social fathering role, contributed to the gender socialization of the boys in their soccer clubs. Their strategies included forming supportive relationships with the boys themselves, as well as creating alliances with parents and professionals to address gendered issues related to respect, sexual and reproductive health (SRH), alcohol and drugs, and involvement in criminal gangs.

## **Coaches’ perceptions of adolescent boys’ problems**

The coaches identified three main areas of problems and underlying dynamics associated with these problems. These were social and behavioral problems related to substance abuse and gangsterism; emotional problems related to family dynamics and home stressors; and class differences related to socio-economic background.

### Social and behavioral problems boys were facing

In the interviews, almost all the coaches said some boys in their clubs were experiencing serious social and behavioral problems, which included the use of illicit drugs, harmful use of alcohol, and involvement in gangsterism:

I have two boys in the team that were involved in drugs … and they haven’t even reached 15 [years of age]. But I was able to help them because I met with their parents. But some of them decided to stop playing football … because if he came across me, he would run away. Jah, so those are the challenges that we come across here in the township. (Bafo, Gugulethu, Township)

Where I am staying, Ward 702, it’s bad and they [boys] know that I am always fighting with them to join football clubs. I have two boys that I have taken out of that [smoking drugs] like there is a boy who was smoking [drugs], and I took him to the [soccer] field. I tried to speak to him, and he is very good, but he was busy hanging out with those who were using drugs, so that’s how our society is, it’s always about having a smartphone, having the *amapiano* [youth pop culture] hats and it’s not just any hats, it’s a symbol that shows that you are in this lifestyle, and you must be recognized. (Ongama, Mthatha, Township)

Some coaches spoke about their efforts to discourage adolescent boys from being involved with criminal gangs, in their townships:

So, I took [gangster] boys from the township and mixed them with boys in my club and after all that effort other coaches were asking how I did that? And I can say after that [intervention], the issue of gangsterism has just disappeared, … Even though I aimed to fight the use of drugs as it was an issue in the community and I decided to target those who are known as drug users to form their own team, and I would sometimes ask them to come and play with us [and that] if they win they will get the money, but I knew that that will not happen, instead we would buy [soft] drinks and bread because smoking drugs won’t take them anywhere…(Tony, Gugulethu, Township).

According to these coaches, the boys’ ‘difficult backgrounds’ and ‘challenging lives’ were linked to the social problems they were facing. Some coaches felt that the problems adolescent boys experienced were starting much earlier in their lives:

You know, [years back] you would never struggle to find 12-year-olds and 14-year-olds to join your club. But now [problems] start from 13 years; nowadays they start the stage and have girlfriends and bunk [be absent from] classes and do all those things. You will find that these things used to happen among 17 or 18-year-olds, but now they start at a younger age, but from 17, 18, and 19, they are already ruined. (Masixole, Mthatha, Township)

There were other aspects of gender socialization that the coaches felt were unhealthy for boys. Most coaches, in both Mthatha and Gugulethu, identified traditional male circumcision (TMC) as one of the key mechanisms for socializing boys. However, several of the coaches believed that the problematic behaviors of adolescent boys could be related to changes in the practice of this rite of passage to manhood. They felt that TMC presented a threat to healthy adolescent development and behavior. They argued that it no longer performs the positive, socializing role that it did in the past.

For the coaches, the changes they were observing in TMC processes, compared to earlier times, were producing poor social and health outcomes for newly circumcised men (*amakrwala*). Specifically, they highlighted the introduction of new practices (e.g. use of illicit drugs, alcohol abuse by initiates, and the ‘Numbers gang’ language) into the custom. Consequently, the coaches felt that amakrwala were attaching new meanings to being a traditionally circumcised man and were behaving differently from what is expected of them:

You see my brother, the initiation schools play a vital role to model these boys, where a boy comes back as a man. But also it plays a bad role because there was a boy who went to initiation school he came back drinking alcohol, but he was not drinking alcohol and not smoking before going to the initiation school, but now during his initiation school days, he was being taken care of by a man who was smoking weed [marijuana] and drinking alcohol, so he had to take those things and feel it’s goodness. After he came out [of the initiation school], he was no longer even going to school. (Masixole, Mthatha, Township)

Another thing that I wrote down is the initiation. You raise a child well, make him play soccer. He goes there [initiation school] and he is told to smoke, “A man smokes”. They drink [alcohol] there, so the child comes back in a worse condition. These are the challenges that we as coaches come across in coaching these adolescent boys. (Bunono, Mthatha, Township)

Having perceived that TMC was no longer performing its intended role of socializing boys into becoming responsible and respectful men, several coaches said that this motivated them to actively intervene in the lives of the boys as an attempt to positively shape their behaviors and masculinities.

### Emotional challenges faced by boys

The coaches also identified underlying emotional problems experienced by the adolescent boys, which they related to the absence of positive father figures and other stressors in the home environment:

His dad separated from his mother when they were young so all he needed was a father figure next to him someone who will guide him. So I said to his mother, “A child must be close to other children and because he was wearing old torn clothes, so if you have a problem send money to me then I will accompany him to buy clothes”. So, all he was lacking was a father figure, someone who would relate with him. So, we continued with this boy, now he is doing Grade 12. He is updating me every time about his things. (Xola, Mthatha, Township)

Similarly, many other coaches perceived many boys as needing a father figure in their lives. On recognition of this need, they felt it was their responsibility to step into the social fathering role. While not all the coaches said they played a social fathering role, most gave accounts where they actively played this role. Masixole’s narrative evidences this:

The [biological] father loved that boy, he was supportive and made sure that his children had everything they need although he was drinking a lot of alcohol. The father got sick and died, after his death, things started to get worse for the boy and the boy was 16-17 years old. The boy would go to school and come back very late even on the training ground he was always late. So, his mother reported the boy to me that he once came back from school drunk. Then I tried to play a father’s role to the boy, and I discovered that there is a challenge that the boy had some bad friends from another township that we had chased from the team because of misbehaving. (Masixole, Mthatha, Township)

For the coaches, taking up the social fathering role was important as they perceived it as filling a critical gap in the lives of the boys, which was resulting in emotional issues and behavioral problems for some boys.

Also, most of the coaches told us that some of their boys came from unstable, conflictual, and punitive home environments and were experiencing enduring emotional problems due to hardships associated with these family situations. For example, witnessing fights between their parents, family dissolution, being physically beaten or deprived of food at home, and other hardships were common at home:

I still remember another boy, his parents were on separation, he was staying with his father, the father was working and then the mother was busy with her life that is not stable [having an extra-marital affair]. There were times when the mother would come to the house and there would be conflicts between the parents where one would claim that the car is theirs, and the mother would come with the boyfriend who is not the boy’s father. (Masixole, Mthatha, Township)

The issue that these boys have come to us [coaches] for, is the situation in their homes. Some boy reported that his father is beating him every now and then, even when he has done nothing wrong… and we would try to engage and assist them. Sometimes we would go to their parents and ask what is happening because we notice that he [boy] is no longer the person he used to be in the field, and we don’t want to continue like nothing is happening … We make sure that we let them [parents] know that we are not trying to teach them ‘how to treat your child but if you are punishing him by not giving him the food, he will go and seek that outside in the streets’. (Thobani, Mthatha, Rural village).

Masixole narrated a story of a boy in his club who was abused by his father and deprived of food as a punishment for not fixing cars:

I still remember in the early 2000s … there was a boy called Funda. That boy was messed up by his father. His father was fixing cars … so when returning from school Funda was expected to fix the cars before he could go to the gym. He had no soccer boots and would steal other children's belts so that he could sell them to get some cash, so when you asked him why he steals, he would say “it's not easy at all, his father is not giving him food if he didn't repair any car”. The boy was 19 years old and he ended up being a criminal and was shot by police officers and died. (Masixole, Mthatha, Township)

Masixole said he had tried to intervene with the boy to stop his criminal behavior, but that the boy explained that his behavior was caused by his difficult experiences at home:

So, you will find out when you met him and talked, he would say “no brother you know my situation. After my mother died, I had to take care of myself, even my brothers do not care about me. So, for me to eat, I must steal”. (Masixole, Mthatha, Township)

### Class spectrum in townships

The third area of underlying problems was class differences due to different socio-economic backgrounds; with a class spectrum that could be narrow or wide depending on the setting. The coaches described how the socio-economic backgrounds of the boys they trained differed, with some boys coming from disadvantaged backgrounds, while some were coming from relatively well-off families:

So it’s not easy to say all I’m focusing on is football… so I need to have an eye on other things apart from football, as a result, I created an emergency fund so that if we are going to a match I put an R100 [∼US$ 6] aside for them to eat after the game. We buy bread and drinks so I can know that if a child came from home without eating, he will come back from the soccer game having eaten something. Unfortunately, the area I reside in has a mix, we’ve got [names of different townships], and [another township], some of the [latter township] boys come from poor homes but if you consider [resource-poor townships], you see all those who come from that side you can tell even by their clothes that ‘hey here there’s a shortage of something’. (Xola, Mthatha, Township)

The coaches’ narratives not only illustrated the class spectrum among the boys but also suggested how this spectrum prompted them to adjust the way they responded. Most coaches perceived boys coming from well-off and stable families as being less vulnerable to most of the problems described earlier. They perceived boys from financially impoverished and unstable family environment as more prone to these problems, and tended to give the latter boys more attention and support:

And it's a vice versa, he [another coach] was close to rich players and I was close to poor players, so that was the reason why his team was not consistent because he was not able to protect these [poor] boys. (Masixole, Mthatha, Township)

In recognition of these class-based differences among the boys, most coaches talked about putting more effort into making the poorest boys feel welcome and comfortable in the clubs, availing themselves more emotionally to them while also providing them with material support, whenever there was a need:

Anyway, I saw it [emergency fund] is needed by the time I was paying for this boy at Vukuzithele Senior Secondary School because he was going to be expelled there, they wanted R500 [∼US$ 30] so I said they must not expel him from school, I took this money and paid… (Xola, Mthatha, Township)

On the other hand, the coaches perceived the boys who were well-off as not needing much support beyond being trained for soccer, even though they tried to form a good player-coach relationship with these boys. For these coaches, it was essential to maintain a good balance in how they treated the boys from the different ends of the class spectrum, for the club to function well.

## **Social Fathering and Gender Socialization**

Our analysis shows that the social and behavioral problems experienced by boys were not straightforward and dealing with them was difficult for the coaches. Yet, most coaches mentioned that, through their clubs, they ended up doing a great deal of work to help socialize adolescent boys and to positively shape their practices and support them in constructing alternative masculinities. In their work of socializing adolescent boys, they employed various strategies including creating an alliance with parents and professionals.

### Coaches as Social Fathers

When the coaches looked at the problems and challenges experienced by some boys in their clubs, one of the most common reactions was to give themselves the responsibility of playing a social fathering role to these boys:

So that makes me think the attention we [coaches] are giving them [boys], they do not get it at home, so we are no longer just coaches we are also the fathers, like for instance when a boy says they do not have food at home so you have to assist where you can … so we have to help because we know the situation at home, so it’s not just coaching it’s nurturing and also fathering the boys. (Thobani, Mthatha, Rural village)

You sometimes find that their [boys’ socio-economic] levels are not the same. But then you realize that the boy [from a disadvantaged background] likes soccer. So, you then start realizing that you are acting as a parent to this boy. (Bafo, Gugulethu, Township)

For the coaches, playing this role was a significant intervention to help the boys change their journeys and think differently about their lives; to encourage them to change their behavior and practices, and for the coaches to mitigate the risk for poor health outcomes in the lives of these boys:

I saw an opportunity, because they had chosen to play soccer than to be standing outside the shops and smoking weed [marijuana], so I thought if I don’t do anything about this, to nurture and groom them through football then we will lose them, and they will end up going to do things that aren’t right. So, I ended up telling them that “as of today you will be playing for my club … I will take you through playing football and we must know that playing football is not the only important thing, the other things that are important are respecting other people, education, working hard and taking life seriously”. (Thobani, Mthatha, Rural village)

I remember we had boys called Fox and his brother Thulz, their big brother was into drugs and was a criminal, so you’d find them [players] with stolen things, but they got out of that life because we [coaches] were close to them, we used to talk to them not to get involved in that life. (Masixole, Mthatha, Township)

In playing the social fathering role, the coaches served as role models to the boys in their clubs by behaving appropriately in their presence, including not drinking alcohol or smoking marijuana.

I am not a person who behaves badly in the area, I don’t swear at anyone… For me, it’s not a show that I would walk in the street carrying alcohol. If I am at home with my friends we just sit and chill in the car, we don’t go out to show people that I can also do these things [drink alcohol]. So even if I do those things, I do it out of my boys’ sight, you get me? So, when I talk to them, I ask if they have ever seen me walking around carrying beer and they say no. So, I say “in the future when your mother sees you carrying a beer, where will you say you learned that from?” And that is a question they cannot answer. (Ayanda, Gugulethu, Township)

Other coaches more deliberately engaged in gender socializing work with the boys as they spoke with them about ‘how a man is expected to behave’, including the need to respect women:

They [boys] don’t know what it means to be a man and how a man should behave. So that is why we try to teach them on a daily basis that they must be respectful not just respecting themselves but respecting the next person and women and this thing of calling women names must end. They must also respect their parents, they must always be clean, we are trying to instill the values, not just about the football, they must always remember what he was taught at the club. And we don’t want anyone to drink or smoke in this team, so we are trying our best to make them good men. (Thobani, Mthatha, Rural village)

The data reveal that the coaches mostly had a hierarchical authority role in the clubs. However, their relationships with the boys also appeared to be more dynamic and less structured than expected and often did not fit the assumed rigid and neatly arranged relationship of the soccer coach-player. While some boys, especially those from financially and socially stable home environments, tended to forge simple relationships with the coaches which involved less nurturing by the coaches, boys from poor families or dysfunctional homes appeared to allow for a more emotionally involved and nurturing relationship that went beyond coaching:

So, there are situations where you see a child’s background and wonder why he behaves the way he does. You then realize that he is missing a father figure or his parents divorced, so we [coaches] have to intervene in such cases and play a father figure and even here on WhatsApp they would take my profile pictures and post them on their walls and say this is my father and when they chat together they would say ‘my life changed ever since I met him, he is a coach, he is a motivator and a spiritual father’, so you would see that indeed he missed that father figure in his life… (Ongama, Mthatha, Township)

The relationships with boys from financially impoverished and unstable family environments almost always extended to the “private space” of home and deeper involvement (e.g. in disciplining methods and schooling) by the coaches. Yet, as we show in the sub-theme below, these relationships were largely not formally agreed to, causing coaches to be uncertain how involved they should be in the boys’ lives, and sometimes their involvement was unwelcomed by parents.

### Social fathering and the family at home

The social fathering role did not replace the boys’ relationships with their family but existed in parallel to those family relationships. Coaches described several ways in which they tried to work with parents, and form alliances with the boys’ parents so that they could work in synergy to shape the boys’ behaviors, masculinities, and lives. This is best illustrated by Coach Ongama:

Our academy has parent leaders called the 12th player with a WhatsApp group where parents report and discuss anything concerning the players. (Ongama, Mthatha, Township)

The coaches shared that while not all parents were eager to be in alliance with them, some parents were willing to work with them in addressing their children’s challenges:

Yes, because now there are a lot of diseases and they [boys] don’t know about that, so these are the challenges that I am facing, I will have to address all these things and discuss them with the parents because at the end of the day I don’t want them to say I am teaching their kids about dating, so we need to talk to these kids together. (Tony, Gugulethu, Township)

… and remember the parents have children and we have players, so a parent does not have a player in their house, so you as the coach you have to go to the boy’s home and build that relationship with the parents so that there could be a smooth way of working together. (Ongama, Mthatha, Township)

While most coaches said parental involvement in clubs was rare, a few coaches mentioned that some parents were involved, had the interest to know what happens in the clubs, and were keen to work in tandem with the coaches in socializing the boys and shaping their behavior.

There was a boy in my club, he became involved in gangsterism at school taking people’s phones, so his mother, as a way of punishing him, said he must stop playing football, and I said to the mother ‘mother please do me a favor, just give me a chance with this boy, then I’ll put him in a programme then if he is not responding to the programme, you can take him … then I sat down with this boy and I showed him that he won’t be involved with people that are using drugs, ‘if you want to grow or want to be independent just like your mother or your father you need to start at school and prioritize school’, and I realized that counseling should be involved in this …, so we continued with this, and you won’t believe he is doing Grade 12 now…(Xola, Gugulethu, Township)

The coaches said that they observed behavior change in some boys, including constructing health-enhancing masculinities, when they worked closely with their parents. However, many coaches had difficult relationships with the parents of some boys. These coaches talked about sometimes being perceived by parents as encroaching into spaces that are not only private but also out of bounds for them. Indeed, the coaches described instances where their attempts to involve themselves in socializing the boys, beyond ‘just training them soccer’, brought tensions in some families:

I still remember some guy called Fuzo who is the boy’s uncle, he came to me with the boy’s father, and they asked me why the boy is no longer in the soccer club, I told them all the story that we argued with the boy’s mother and when I punished the boy, the mother said I was harsh. The punishment back then was that when the team is going to play the person being punished will have to take the soccer kit home and wash it, so the mother was not happy with this, so I ended up stopping with the punishment. (Masixole, Mthatha, Township)

Consequently, while these coaches felt compelled to involve themselves in socializing the boys, they recognized they were constrained in doing so by the tensions their involvement brought, disapproval by some parents, and subtle feedback from some parents who felt the coaches were ‘going too far and involving themselves in matters that do not concern them’.

Indeed, most coaches shared their frustrations regarding the difficulties they encountered in navigating these dynamic relationships with the parents, which included having to contend with parents who subtly questioned their intentions, and even seeing soccer as a distraction from other duties.

We [coaches] are trying to assist the parents in raising them [boys] through what they love, which is football. I would say, some parents say football is destructing their children and that it is the reason they do not look after the livestock and the reason they come back home very late, and those kinds of parents are the ones who say I am trying to make money using their kids. (Thobani, Mthatha, Rural village).

One parent reportedly felt that soccer interferes with the boy’s academic studies:

His [player’s] mother called us [coaches] and she said she feels like this boy is being disturbed by playing soccer… she said she will not buy him soccer training shoes this year, the boy must just study. We tried to explain that we feel like he is not studying when he is at home, and we requested that he can at least go to soccer training once a week and study the other days, … she was not getting it, she wanted the boy to attend extra classes, and said she wants to monitor that herself. We never did much to convince her that we can do this through the club, go an extra mile, and find him a teacher who can provide him with academic support, we never did because we saw from her response that she was not open to working with us. (Ongama, Mthatha, Township)

Social fathering was thus a common, but complicated role for coaches to take on, one that required constant and sometimes complex negotiation with parents and other family members about how best to support the boys in their club.

### Professional support at the limits of social fathering

Coaches also described confronting critical limits to their (or the boys’ families) ability to fully support their club members. In many cases, for example, coaches felt like they didn’t have enough information to enable them to play this supportive fathering role effectively:

So, but I promise you from now on we will try to be open to them [boys], because of the way you have questioned me. We will try in any way to be open to them; it’s just that we are not qualified for these things [SRH issues, sexuality, and dating], and it becomes a problem how to engage in a conversation with boys concerning these things. As black people sometimes there are things that you are afraid to talk about. (Bafo, Gugulethu, Township)

Coaches spent a considerable amount of time with the adolescent boys, especially during the soccer season, and they were able to get to know some of the personal and social challenges faced by individual boys. This deeper level of interpersonal engagement is what appears to motivate the coaches to take on a social fathering role. Yet most coaches felt that they were unequipped to manage this role, with some feeling they needed more help from health professionals:

It was a team of under-17s that was playing very well and got promoted, but we would only have four of them attending training … it was *amapara* [illicit drug users] and they would come to practice smelling of dagga and intoxicated with drugs. It was such an unfortunate situation…you see? So, if you are not a psychologist or there is no psychologist to help you, then you won’t know what to do, and you don’t get any cooperation from the parents because they don’t have a passion for their kids to play soccer, you see? (Viwe, Mthatha, Rural village)

A few coaches talked about using professionally trained people to engage the boys in matters that they felt were beyond their capabilities. These coaches reportedly had a standing agreement with some professionals or institutions to come to their clubs at certain times and address issues affecting adolescent boys:

We have a medical doctor, XXX, who assists with the medical side of things. We also have a psychologist, XXX who comes once a month and have sessions with boys who need it. For example, when there is a player you see is not from a good background, or who is not free around other players, you notice that there is something you as a coach you do not understand, then we call him in those instances. He works with XXX, who is a life coach. She deals with how they [boys] should conduct themselves in life and motivates them. (Ongama, Mthatha, Township)

Coaches like Bafo indicated that they lacked the capacity to address issues related to gender, sexual reproductive health, and dating and needed to be trained on these issues to be able to adequately address them:

Even when it comes to girls there are those [boys] who are dating, but as I have said previously, we still need some training because we are not well trained. We don’t realize other things until it is too late. (Bafo, Gugulethu, Township)

Another coach asserted that it would not be difficult to engage boys from his club on sexual and reproductive health (SRH) issues affecting them, yet he thought this was beyond his core function as a coach and that he would prefer to be assisted by a health professional in addressing these issues:

My engagement with the boys is mainly about soccer – and the boys only perceive me as a soccer coach; …my language to the boys is largely about soccer’. As such, I feel that as part of the strategy to engage the boys about health issues including sexual and reproductive health and sexuality issues affecting them, someone who understands health issues/challenges affecting boys would need to come and talk with the boys. (Thabo, Gugulethu, Township)

There could be various reasons the coaches preferred to work with professionals in engaging boys in these issues. First, they may have perceived it as a viable strategy to connect the boys to significant resources, but also draw support for themselves as they had limited capacity to address all the challenges on their own. Alternatively, the coaches may have felt overwhelmed and/or underqualified to address these issues, hence reaching out to those with expertise to support them.

# **Discussion**

In this study, we explored how gender socializing works in soccer clubs, specifically assessing the potential of soccer clubs as approaches for gender transformative socialization of adolescent boys. We also explored the degree to which such socialization involves a social fathering role played by coaches, and how that may have opportunities for having gender transformative elements in that socialization.

Broadly, the findings of this study provide useful insights into how gender work with adolescent boys can be done within soccer clubs. We presented findings that suggest that soccer clubs are potentially valuable spaces for socializing gender transformation, and social fathering is an important component of that if it is going to be successful. Specifically, we have demonstrated that soccer clubs and coaches can be significant socializing spaces and agents, respectively, for adolescent boys in Black South African communities. Moreover, we have shown that soccer coaches were eager to and do play the social fathering role, yet playing this role is complicated. They faced challenges with playing a social fathering role related to balancing the role of coach and social father in the relationship with the boys, limited knowledge on how to address problems, and difficult relationships with parents. They outlined various strategies to address the multifaceted social, emotional, and health problems they perceived among boys, especially those from poorer and dysfunctional families. Key strategies were the alliances the coaches forged with the parents and professionals. Our findings clearly show that social fathering is complex and that it needs support, resources, training, and skills. Moreover, it requires the engagement of others as the coaches cannot do it all by themselves.

Through playing the social fathering role, the coaches actively intervened when the boys were engaging in destructive or risky behaviors (e.g. alcohol and drug use or engagement in gangsterism) and masculinities. These findings corroborate Medich et al.’s (23) findings that coaches in Cape Town townships played the roles of a father to boys in their clubs by giving them advice and guidance on how to address the challenges they were facing, including advice on avoiding involvement in gangsterism. Our findings show how the coaches’ interventions happened through educating and supporting (emotionally and socially) the boys to alter their journeys. They challenged the boys to critically reflect on their risky behaviors and practices, approach life in a more progressive way (e.g. work hard at school, and be respectful towards women), think differently about their [difficult] life circumstances, and set new progressive life goals. Furthermore, some coaches more directly engaged in gender work with the boys, challenging destructive masculinities and gender norms. We contend that this gender socializing has the potential to mitigate the risk for poor health outcomes for boys. We also contend that with the right support and resources, the social fathering role played by these coaches has the potential for gender-transformative socializing as well, to support the development of more equity-based gender norms. To achieve this transformative gender socializing, the coaches would need to be more actively supported by the parents of the boys and by professionals and broader community support organizations.

Soccer is popular in most Black African communities in South Africa (40) and soccer clubs are considered vital for socializing adolescent boys and young men. In our study, a key factor that motivated the coaches to get involved in soccer clubs and play the social fathering role was the widespread perception that some of the other ways of socializing adolescent boys (e.g. TMC) are failing (41, 42). Historical literature from South Africa suggests that in the past TMC succeeded in its intended role of socializing young boys into local norms of respectful, responsible men because of certain elements it possessed (43, 44). Hellman (45) has noted that, in the past, TMC in Black rural communities was more of a group process that enabled a sense of an age cohort ―with initiates positively influencing each other, yet in more recent times, TMC has largely become an individual process. Secondly, scholars like Wilson and Mafeje (46) observed in Langa township, Cape Town, that in contemporary times, the emphasis is put on the clinical operation (circumcision), with very little focus on the educational aspect of the custom (see also 47). Moreover, Wilson and Mafeje (46) observed that in more recent times the period of seclusion for initiates is shorter, thus limiting the time for the initiates to be educated on particular attitudes, moral values such as self-respect and respect for others, and social responsibility.

Owing to their perception that TMC was no longer performing its intended socializing role, the coaches took on the responsibility of socializing adolescent boys, with many becoming social fathers to boys in their clubs. However, despite their eagerness to play this role, doing so came with several complex challenges. First, some coaches felt they lacked the capacity to play this role, citing a knowledge gap on the approaches for addressing the social and health problems adolescent boys face. Second, for some coaches, playing this role resulted in them having hostile relationships and conflicts with the families of some boys, as the families felt that the coaches were overreaching or overstepping the boundary. However, our findings suggest that some boys reportedly appreciated the social fathering aspect of their relationships with the coaches, with some publicly (through social media platforms) acknowledging the coaches as father figures to them. This finding reflects that reported in a study among young Black men in informal settlements in Durban who said they cherished the ‘guidance on growing up’ that they received from social fathers in their communities (49). We argue, however, that it is unreasonable to expect coaches to do it all and play all these fathering roles to boys in their clubs. While all the coaches were prepared to play this role, a lot of them felt they lacked the capacity to do so, and that they needed more skills and support. Because of their inadequate skills, knowledge, and training, they reached out to parents and professionals to try to fill these gaps.

Our findings suggest that a typical model for a coach, in the study settings, is that they become social fathers who provide guidance, discipline, affection, and emotional and social support to adolescent boys in the clubs. While this makes coaches important father figures to some boys, a more salient finding is that the coaches catalyze new relationships for the boys. They become brokers for new alliances and relationships between the boys and their parents and professionals, which, in turn, become useful sources of support for the boys to address their social and emotional challenges. We have presented a finding showing that in one club the coaches formed a WhatsApp group with the parents of the players which allowed the parents to engage with the coaches about social and behavioral challenges they felt their boys were encountering and work in tandem with the coaches in finding solutions to these problems.

There is a growing body of literature in South Africa focusing on soccer clubs as spaces for gender work (23, 25). For example, Medich and colleagues (23) assert that ‘soccer is a facilitator for friendship bonding, for discourses of masculine agendas, and for larger discussion on gender dynamics’ (p.196). Our findings add to this body of knowledge by showing how soccer coaches engaged in the process of gender socializing young boys in resource-limited communities in South Africa. In our study, the coaches emphasized to the boys the significance of self-respect, respect for women and elders, discipline, and positive contribution to society. We argue that through such gender work, the coaches were intending to shape and reinforce a positive soccer masculinity (49) among the boys, one that will make both their coaching careers and boys’ soccer careers successful. However, in engaging in this gender work, it is unclear whether the coaches had made a conscious effort to shape a new gender-equitable male youth. This warrants further exploration through targeted in-depth research on the coaches’ role and motivations for engaging adolescent boys in sports.

We have presented a finding that supports South African research that has shown that work on gender is already happening in soccer clubs in Black communities (23). We, therefore, argue that soccer clubs present an opportunity for researchers and programmers to use them as vehicles for behavior change (51) by leveraging the gender work that is already occurring in these clubs, and ensuring that such work is informed by progressive theories of gender, power, and masculinity. Our findings also suggest that soccer clubs offer a useful entry point to draw adolescent boys away from social vices (e.g., using illicit drugs and involvement in gangsterism), yet we contend that to succeed in re-socializing adolescent boys into health-promoting norms of gender, there is a need for structural changes that incorporate a focus beyond sports. Specifically, in South Africa, there is an urgent need for structural interventions aimed at improving the livelihoods of working-class people in resource-poor communities. Most Black South African communities are characterized by high levels of poverty, unemployment, and dysfunctional families (51). Thus, our finding on absent or uninvolved fathering in the families of some boys in soccer clubs should be understood in the context of economic disenfranchisement of Black people which has its roots in colonial and apartheid systems (52), and the continued socio-economic, socio-political, and structural forces in democratic South Africa that continue to adversely affect and shape the lives of adolescent boys in poor Black communities.

**Implications for programming and research**

Our findings highlight both the practice of soccer clubs and coaches as avenues for gender socialization of adolescent boys that is aimed at promoting their health and social well-being. It provides an opportunity to reflect on how these same gender socializing mechanisms could be supported as ideal opportunities for transformative gender socializing. To achieve this, soccer clubs and coaches may benefit from working closely with and drawing support from existing NGOs, and sport and youth development initiatives that provide gender-transformative programmes and parenting support interventions.

Research is needed to explore the actual gender politics of the coaches and in their interactions with adolescent boys. As we have shown, gender work is already happening within soccer clubs, yet whether that work reinforces conservative gender norms and masculinities warrants further research. The notion that soccer coaches can play a social fathering role as part of gender-transformative interventions is a key assumption that requires further examination of the kind offered in this paper. To strengthen this evidence base, future studies would do well to interview adolescent boys in soccer clubs as that would add an important dimension to our understanding of both the gender socialization happening within soccer clubs and how this can be shaped to be gender transformative. Last, there is a need to understand the longer-term impact and sustainability of soccer clubs as potential gender-transformative vehicles, and what specific aspects of gender socializing might be helpful or harmful for the health of adolescents.

### **Strengths and limitations**

The scope of our study is limited on the question relating to the ultimate boy-product the coaches wanted to see when they engaged in the social fathering role with the boys in their clubs. As such, our data do not allow us to adequately address this question in this paper. Future studies exploring the social fathering role played by soccer coaches and how it may work to socialize boys into a gender order in a particular setting would do well to explore this question. Exploring this question is critical as it lies at the heart of transformative gender work with adolescent boys.

The purposive selection of coaches may have been more biased towards coaches that were more actively inclined towards supporting socializing of their soccer team members, and the transferability of the findings may be limited to similar settings. The strength of the study is that it included coaches from urban and rural settings which gives it broader transferability. Coaches provided a nuanced view of their experiences, including being open about their challenges, and this together with the communalities across the two settings, increases our sense of the credibility of the findings. We hope these findings would be used to generate hypotheses for future research with adolescent boys in soccer clubs in South Africa and elsewhere.

### **Reflexivity**

The authors are researchers, who are situated within both academia and practice. Two of the authors were born and had lived in the two settings of this study, as adolescent boys, and amateur soccer players (YS and MM) and were able to provide an insider perspective on this topic. Their prior experiences in these settings may potentially bias their views on the analysis of the data. Other authors (CC, NL and ML) are White academics who have lived for many years in the Western Cape province where they conducted extensive health systems research, community development work, health promotion work, and gender transformative interventions with boys and men in Black poor communities in the Western Cape province, including Gugulethu. As such, while they have extensive knowledge on this topic and are highly skilled in qualitative data analysis and interpretation, their personal experiences, identities, and social positions may have influenced how they interpreted the data. Notwithstanding, the discussion and refining of interpretations across the author team ensured that the findings and interpretations reported here were grounded in the data collected in the study.

# **Conclusions**

In Black African communities in South Africa, families are viewed as vital spaces where boys get gender socialization. However, in resource-poor communities, like the settings of this study, there is a perception that the conventional strategies of socializing adolescent boys have been compromised and are failing in this role. Gender socialization of boys can be done either conservatively or progressively, and soccer clubs represent an opportunity for the latter approach, as we have shown here that gender work that is health promoting and produces a soccer masculinity among adolescent boys is already happening in clubs, and that this could lay the foundations for gender transformative work. However, coaches need to be capacitated and supported to ensure that gender work within their clubs is progressive.

In this paper, we have shown that, in the settings of our study, soccer coaches play an important role in socializing adolescent boys, but we contend that they cannot do it all by themselves. They need training, material, and support. We need to think about the coaches’ role as being that of helping adolescent boys build networks and relationships with other people as well, from which the boys can draw to address their social and health challenges.

# **Acknowledgements**

The authors would like to thank the participants who shared their stories, insights, and experiences with us.

# **Author Contributions**

**Conceptualization:** Yandisa Sikweyiya, Christopher, J. Colvin.

**Data curation:** Yandisa Sikweyiya, Christopher, J. Colvin.

**Formal analysis:** Yandisa Sikweyiya, Christopher J. Colvin, Natalie Leon, Mark, N. Lurie, Mandla Majola.

**Funding acquisition:** Christopher J. Colvin, Mark, N. Lurie.

**Investigation:** Yandisa Sikweyiya, Christopher, J. Colvin.

**Methodology:** Yandisa Sikweyiya, Christopher, J. Colvin.

**Project administration:** Yandisa Sikweyiya.

**Supervision:** Christopher, J. Colvin.

**Validation:** Christopher, J. Colvin, Natalie Leon, Mark, N. Lurie.

**Writing – original draft:** Yandisa Sikweyiya, Christopher, J. Colvin.

**Writing – review & editing:** Yandisa Sikweyiya, Christopher, J. Colvin, Natalie Leon, Mark, N. Lurie, Mandla Majola.

## **Disclosure statement**

No potential conflict of interest was reported by the authors.

# **References**

1.	Gibbs A, Vaughan C, Aggleton P. Beyond ‘working with men and boys’:(re) defining, challenging and transforming masculinities in sexuality and health programmes and policy. Taylor & Francis; 2015.

2.	Ruane-McAteer E, Gillespie K, Amin A, Aventin Á, Robinson M, Hanratty J, et al. Gender-transformative programming with men and boys to improve sexual and reproductive health and rights: a systematic review of intervention studies. BMJ Global Health. 2020;5(10):e002997.

3.	WHO. Gender mainstreaming for health managers: a practical approach: Geneva: world Health organisation; 2011.

4.	Jewkes R, Willan S, Heise L. Effective design and implementation elements in interventions to prevent violence against women and girls. Pretoria: South African Medical Research Council. 2020.

5.	Flood M. Men’s Antiviolence activism and the construction of gender-equitable masculinities. Alternative masculinities for a changing world: Springer; 2014. p. 35-50.

6.	Salazar M, Öhman A. Negotiating masculinity, violence, and responsibility: A situational analysis of young Nicaraguan men’s discourses on intimate partner and sexual violence. Journal of Aggression, Maltreatment & Trauma. 2015;24(2):131-49.

7.	Colvin CJ. Strategies for engaging men in HIV services. The Lancet HIV. 2019;6(3):e191-e200.

8.	Flood M. Work with men to end violence against women: a critical stocktake. Culture, Health & Sexuality. 2015;17(sup2):159-76.

9.	Dworkin SL, Fleming PJ, Colvin CJ. The promises and limitations of gender-transformative health programming with men: critical reflections from the field. Culture, health & sexuality. 2015;17(sup2):128-43.

10.	Gittings L, Hodes R, Colvin CJ, Zungu N. Things less spoken-HIV research with adolescent boys and young men: Implications for theory, policy and practice. 2020.

11.	Ratele K. Working through resistance in engaging boys and men towards gender equality and progressive masculinities. Culture, Health & Sexuality. 2015;17(sup2):144-58.

12.	Gibbs A, Washington L, Abdelatif N, Chirwa E, Willan S, Shai N, et al. Stepping Stones and Creating Futures intervention to prevent intimate partner violence among young people: cluster randomized controlled trial. Journal of Adolescent Health. 2020;66(3):323-35.

13.	Jewkes R, Nduna M, Levin J, Jama N, Dunkle K, Puren A, et al. Impact of stepping stones on incidence of HIV and HSV-2 and sexual behaviour in rural South Africa: cluster randomised controlled trial. Bmj. 2008;337:a506.

14.	Maina BW, Orindi BO, Sikweyiya Y, Kabiru CW. Gender norms about romantic relationships and sexual experiences among very young male adolescents in Korogocho slum in Kenya. International journal of public health. 2020:1-10.

15.	Kågesten A, Gibbs S, Blum RW, Moreau C, Chandra-Mouli V, Herbert A, et al. Understanding factors that shape gender attitudes in early adolescence globally: A mixed-methods systematic review. PloS one. 2016;11(6):e0157805.

16.	Blum RW, Mmari K, Moreau C. It begins at 10: How gender expectations shape early adolescence around the world. Journal of Adolescent Health. 2017;61(4):S3-S4.

17.	Addis ME, Mahalik JR. Men, masculinity, and the contexts of help seeking. American psychologist. 2003;58(1):5.

18.	Ninsiima AB, Leye E, Michielsen K, Kemigisha E, Nyakato VN, Coene G. “Girls have more challenges; they need to be locked up”: a qualitative study of gender norms and the sexuality of young adolescents in Uganda. International journal of environmental research and public health. 2018;15(2):193.

19.	Bandura A. Social-learning theory of identificatory processes. Handbook of socialization theory and research. 1969;213:262.

20.	Maina BW, Sikweyiya Y, Ferguson L, Kabiru CW. Conceptualisations of masculinity and sexual development among boys and young men in Korogocho slum in Kenya. Culture, health & sexuality. 2022;24(2):226-40.

21.	Maina BW, Izugbara C, Sikweyiya Y, Nandongwa I, Kabiru CW. Gender Norms and Sexual Behavior among Very Young Adolescents in Sub-Saharan Africa: A scoping review. The Routledge Handbook of African Demography. 2022:330-48.

22.	Connell R. The sociology of gender in Southern perspective. Current Sociology. 2014;62(4):550-67.

23.	Medich M, Mindry D, Tomlinson M, Rotheram-Borus MJ, Bantjes J, Swendeman D. The pull of soccer and the push of Xhosa boys in an HIV and drug abuse intervention in the Western Cape, South Africa. SAHARA-J: Journal of Social Aspects of HIV/AIDS. 2018;15(1).

24.	Haudenhuyse RP, Theeboom M, Coalter F. The potential of sports-based social interventions for vulnerable youth: Implications for sport coaches and youth workers. Journal of youth studies. 2012;15(4):437-54.

25.	Swendeman D, Bantjes J, Mindry D, Stewart J, Tomlinson M, Rotheram-Borus MJ, et al. The Experiences of Young Men, Their Families, and Their Coaches Following a Soccer and Vocational Training Intervention to Prevent HIV and Drug Abuse in South Africa. AIDS Education and Prevention. 2019;31(3):224-36.

26.	Herek GM. On heterosexual masculinity: Some psychical consequences of the social construction of gender and sexuality. American Behavioral Scientist. 1986;29(5):563-77.

27.	Connell RW, Messerschmidt JW. Hegemonic masculinity: Rethinking the concept. Gender & society. 2005;19(6):829-59.

28.	Zarrett N, Fay K, Li Y, Carrano J, Phelps E, Lerner RM. More than child's play: variable-and pattern-centered approaches for examining effects of sports participation on youth development. Developmental psychology. 2009;45(2):368.

29.	Fraser-Thomas JL, Côté J, Deakin J. Youth sport programs: An avenue to foster positive youth development. Physical education & sport pedagogy. 2005;10(1):19-40.

30.	Hosegood V, Madhavan S. Understanding fatherhood and father involvement in South Africa: Insights from surveys and population cohorts. Fathering. 2012;10(3):257.

31.	Clowes L, Ratele K, Shefer T. Who needs a father? South African men reflect on being fathered. Journal of gender studies. 2013;22(3):255-67.

32.	Colvin CJ, van Pinxteren M, Majola M, Leon N, Swartz A, Mbokazi N, et al. Fostering a healthy public for men and HIV: a case study of the Movement for Change and Social Justice (MCSJ). Palgrave Communications. 2020;6(1):1-8.

33.	StatsSA. City of Cape Town census data. Pretoria: Statistics South Africa; 2011.

34.	StatsSA. Statistical release P0211: Quarterly Labour Force Survey. Pretoria: Statistics South Africa; 2019.

35.	Wood K, Jewkes R. 'Love is a Dangerous Thing': Micro-Dynamics of Violence in Sexual Relationships of Young People in Umtata. Pretoria: CERSA (Women's Health), South African Medical Research Council; 1998.

36.	Chireshe R, Jadezweni JM, Cekiso M, Maphosa C. Poverty: Narratives and experiences of street children in Mthatha, eastern cape, South Africa. Journal of Psychology in Africa. 2010;20(2):199-202.

37.	Sikweyiya Y, Jewkes R. Force and temptation: contrasting South African men's accounts of coercion into sex by men and women. Culture, health & sexuality. 2009;11(5):529-41.

38.	Braun V, Clarke V. Using thematic analysis in psychology. Qualitative research in psychology. 2006;3(2):77-101.

39.	Dahlgren L, Emmelin M, Winkvist A. Qualitative methodology for international public health: Umeå Universitet; 2007.

40.	Sewpaul V. On national identity, nationalism and Soccer 2010: Should social work be concerned? International Social Work. 2009;52(2):143-53.

41.	Vincent L. ‘Boys will be boys’: traditional Xhosa male circumcision, HIV and sexual socialisation in contemporary South Africa. Culture, Health & Sexuality. 2008;10(5):431-46.

42.	Prusente S, Khuzwayo N, Sikweyiya Y. Exploring factors influencing integration of traditional and medical male circumcision methods at Ingquza Hill Local Municipality, Eastern Cape: A socio-ecological perspective. African Journal of Primary Health Care & Family Medicine. 2019;11(1):1-11.

43.	Ntombana L. Xhosa male initiation and teaching of moral values: An exploration of the role of traditional guardians in teaching the initiates: IKS in other contexts. Indilinga African Journal of Indigenous Knowledge Systems. 2009;8(1):73-84.

44.	Delius P, Glaser C. Sexual socialisation in South Africa: A historical perspective. African studies. 2002;61(1):27-54.

45.	Hellmann E. Problems of urban Bantu youth: report of an enquiry into the causes of early school-leaving and occupational opportunities amongst Bantu youth in Johannesburg: South African Institute of Race Relations; 1940.

46.	Wilson M, Mafeje A. Langa: a study of social groups in an African township: Oxford University Press Cape Town; 1963.

47.	Longmore L. The dispossessed: A study of the sex-life of Bantu women in urban areas in and around Johannesburg. 1959.

48.	SISWANA A. CHAPTER SEVEN RE-CLAIMING THE SIGNIFICANCE OF ULWALUKO INITIATION RITE IN THE EASTERN CAPE, SOUTH AFRICA ANELE SISWANA. Children in South African Families: Lives and Times. 2016:166.

49.	Andrew Gibbs LW, Smanga Mkhwanazi, Sivuyile Khaula, Zama Khoza, Mziwethu Gcuma, Yandisa Sikweyiya. Men's emotions, violence and change. In: Deevia Bhana MC, Peter Aggleton, editor. Sex, Sexuality and Sexual Health in Southern Africa. 1st ed: Routledge; 2022.

50.	Borchardt K, Haber-Curran P. Positive masculinities within male athletic teams and fraternities. Journal of Student Affairs. 2015;24:99-105.

51.	Rotheram-Borus MJ, Tomlinson M, Durkin A, Baird K, DeCelles J, Swendeman D. Feasibility of using soccer and job training to prevent drug abuse and HIV. AIDS and Behavior. 2016;20(9):1841-50.

52.	Gibbs A, Sikweyiya Y, Jewkes R. ‘Men value their dignity’: securing respect and identity construction in urban informal settlements in South Africa. Global health action. 2014;7(1):23676.

53.	Virdee S. Racialized capitalism: An account of its contested origins and consolidation. The Sociological Review. 2019;67(1):3-27.

# **Supporting information**

S1. Interview Guide
